# Association between different anticholinergic drugs and subsequent dementia risk in patients with diabetes mellitus

**DOI:** 10.1371/journal.pone.0175335

**Published:** 2017-04-06

**Authors:** Yu-Wan Yang, Hsin-Ho Liu, Tien-Huang Lin, Hsun-Yang Chuang, Tengfu Hsieh

**Affiliations:** 1 Department of Neurology, China Medical University Hospital Taichung, Taiwan; 2 School of Medicine, China Medical University, Taichung, Taiwan; 3 Department of Urology, Taichung Tzu Chi Hospital, Buddhist Tzu Chi Medical Foundation, Taichung, Taiwan; 4 School of Medicine, Tzu Chi University, Hualian, Taiwan; 5 Department of Research, Taichung Tzu Chi Hospital, Buddhist Tzu Chi Medical Foundation, Taichung, Taiwan; Istituto Di Ricerche Farmacologiche Mario Negri, ITALY

## Abstract

**Background:**

The effects of oxybutynin, solifenacin and tolterodine on dementia risk in patients with diabetes mellitus (DM) remain unknown. We investigated the effects of oxybutynin, solifenacin and tolterodine on dementia risk in patients with DM.

**Methods:**

We conducted a cohort study by using the diabetes dataset of the Taiwan National Health Insurance Research Database from 1 January, 2002 to 31 December, 2013. We included 10,938 patients received one type of oxybutynin, solifenacin, or tolterodine, while 564,733 had not. We included a comparable number of patients not receiving oxybutynin, solifenacin, or tolterodine as controls through systematic random sampling matching by age, gender, and the year of the index date with 1 to 1 ratio. The dementia risk was estimated through multivariate Cox proportional hazard regression after adjustment for several confounding factors.

**Results:**

The dementia event rates were 3.9% in the oxybutynin group, 4.3% in the solifenacin group, 2.2% in the tolterodine group and 1.2% in the control group (P<0.001). The adjusted HRs compared to nonusers of anticholinergic drugs were 2.35 (95% CI, 1.96 to 2.81), 2.16 (95% CI, 1.81 to 2.58), and 2.24 (95% CI, 1.85 to 2.73), respectively, for patients receiving oxybutynin, solifenacin, or tolterodine.

**Conclusion:**

Our study indicates an association between taking oxybutynin, solifenacin and tolterodine and the subsequent diagnosis of dementia in DM patients. Moreover, the patients using oxybutynin had highest risk. The impact of these three drugs on risk of dementia in non-diabetic populations is warrant.

## Introduction

Overactive bladder (OAB) is a common urinary tract condition in which patients experience excessive urination frequency along with a heightened sense of urinary urgency [1, 2]. This condition has a considerably negative quality of life impact, and entails resource-intensive treatment. A high rate of OAB is observed in DM patients [3, 4]. Treatment is commonly pursued using oxybutynin, solifenacin and tolterodine [2, 5].

Generally speaking, new drugs must pass through three stages of clinical trials prior to securing approval for public distribution. This process helps identify most side effects and risks. However, some side effects may take considerable time to become observable, and thus may not be identified in clinical trials before the drug is offered for sale. For example, the risk of pioglitazone for bladder cancer was not discovered during clinical trials, and only became apparent once the drug was on the market [6, 7].

Oxybutynin, solifenacin and tolterodine are all anticholinergic drugs which use muscarinic acetylcholine receptors to reduce spasmolyitic effects on bladder smooth muscle in the treatment of OAB.[8, 9] However, muscarinic acetylcholine receptors are located in the brain, and blocking these receptors may result in the development of neurological diseases such as dementia [10, 11]. Clinical findings regarding the potential for these drugs to cause cognitive impairment remain controversial, with a minority of researchers maintaining that these drugs increase the risk of dementia [12, 13]. DM patients are inherently more prone to dementia, thus particular care must be taken in using anticholinergic drugs to treat OAB in DM patients [14, 15]. To date, very few large-scale studies have examined the impact of anticholinergic drugs on the risk of dementia in DM patients, and this remains an issue which requires proper investigation [5].

Taiwan’s National Health Insurance database provides a comprehensive repository of entire medical history for all of Taiwan’s diabetic patients, making it suitable for use in academic research [16, 17]. This study uses the NHI database to examine the impact of oxybutynin, solifenacin and tolterodine use on dementia risk in diabetics.

## Material and method

### Ethics statement

The Institutional Review Board of Taichung Tzu Chi General Hospital in Taiwan approved the study protocol (REC104-255). Because the identification numbers and personal information of the individuals in this study were not included in the secondary files, the review board waived the need for written consent.

### Data source

This study used the diabetes dataset of the NHIRD from 1 January, 2002 to 31 December, 2013. The NHIRD is maintained by Taiwan’s National Health Research Institutes, and is made available to researchers (http://nhird.nhri.org.tw/date_01_en.html).

### Study design

In our study, the International Classification of Diseases, 9th Revision, Clinical Modification (ICD-9-CM) diagnosis codes and ICD-9-CM treatment codes were evaluated. Defined daily dose (DDD) is a unit for measuring the assumed average daily maintenance dose of a drug consumed for its main indication in adults [18]. The cumulative DDD (cDDD), which indicates the duration of exposure, was estimated as the sum of dispensed DDD of oxybutynin, solifenacin, and tolterodine.

The study design featured a study cohort and a comparison cohort. The dependent variables were diagnosis of dementia (ICD-9 code 290.0, 290.1, 290.2, 290.3, 290.4, 294.1, 331.0) made by a neurologist or psychiatrist. We selected patients who had been newly diagnosed with diabetes (ICD-9-CM code 250.xx) and who were followed up between 1 January, 2002 and 31 December, 2013. We then excluded patients who had been newly diagnosed with dementia before the index date. We also excluded patients who had been diagnosed with dementia less than 180 days following the index date.

We then selected patients who had received oxybutynin, solifenacin, or tolterodine between 1 January, 2002 and 31 December, 2012 as the study cohort and used the date of first drug administration as the patient’s index date. The comparison cohort included all other patients with diabetes who did not receive oxybutynin, solifenacin, or tolterodine. We attempted to reduce selection bias by bundling many confounding covariates that may be present in an observational study with this number of variables; we then implemented a systematic random sampling design to select a matching sample from the comparison cohort frequency matched by age, gender, and the year of the index date. The matching comparison to case ratio was 1 to 1. The flowchart of the number of individuals at each stage of the study was showed in S1 Fig.

The independent variables were co-morbidity (hypertension, lipid disorders, atrial fibrillation, chronic kidney disease, coronary artery disease and heart failure), geographical area of residence, urbanization, and socioeconomic status (SES).

Patients who used oxybutynin, solifenacin, or tolterodine for fewer than 28 cDDDs were defined as nonusers. We also excluded the patients who received more than one anticholinergic drug.

### Other variables

Subjects were classified into two groups by income: (1) low SES: below US$528 per month; and (2) high SES: US$528 per month or more. We selected US$528 as the low income cutoff point because this was the government-stipulated minimum wage for full-time employees in Taiwan in 2006 [19]. Geographic region of residence was recorded as northern, central, southern, and eastern Taiwan. The regions where the individuals resided in Taiwan were classified into 7 levels of urbanization based on 5 indices: population density, percentage of residents with college level or higher education, percentage of residents > 65 years of age, percentage of residents who work in agriculture, and the number of physicians per 100,000 residents [20]. The urbanization level of residences was categorized as urban (urbanization level: 1) and un-urban (urbanization level: 2–7).

### Statistical analysis

We used SPSS version 15 software (SPSS Inc., Chicago, IL, USA) for all data analyses. We also used Pearson’s chi-square test for categorical variables such as SES, geographic region of residence, and co-morbidities. Continuous variables were analyzed using a one-way ANOVA. The cumulative risk of dementia for those who did and did not receive anticholinergic drugs was estimated using Kaplan-Meier survival curves. A Cox proportional hazards regression model adjusted for patient characteristics (age, co-morbidity, SES, and geographic region) was used to analyze the association of anticholinergic drugs with subsequent dementia during the 10-year follow-up period. We calculated hazard ratios (HRs) along with 95% confidence intervals (CIs) using a significance level of 0.05. A two-sided P-value (P<0.05) was used to determine statistical significance.

## Results

A total of 619,216 patients with diabetes were included in our study cohort. Of these, we excluded 3,901 patients who had dementia before the diabetes diagnosis and 10,279 patients who had received oxybutynin, solifenacin, or tolterodine before the diabetes diagnosis or had received more than one kind of oxybutynin, solifenacin, or tolterodine. Of the initial sample, 12,126 had received only one type of oxybutynin, solifenacin, or tolterodine, while 592,910 had not. Matching found 2,540 patients in each group with the same age and gender distribution. The demographic characteristics and selected co-morbidities for these cohorts are shown in Table 1. The patients who did not receive anticholinergic drugs were more likely to be younger and have fewer comorbidities.

**Table 1:** Baseline characteristics.

Characteristics	Before match					After match				
	Oxybutynin	Solifenacin	Tolterodine	Control	p-value	Oxybutynin	Solifenacin	Tolterodine	Control	p-value
Patient no.	4680	4831	2615	592910		2540	2540	2540	2540	
Age (mean±SD)	64±10	62±10	63±11	55±12	<0.001	62±10	62±10	62±10	62±10	NA
Male (%)	3296(70.4)	2821(58.4)	1644(62.9)	329686(55.6)	<0.001	1618(63.7)	1618(63.7)	1618(63.7)	1618(63.7)	NA
Comborbidities (%)										
Hypertension	2171(46.4)	2147(44.4)	1192(45.6)	194209(32.8)	<0.001	1130(44.5)	1098(43.2)	1171(46.1)	1009(39.7)	<0.001
Lipid disorders	695(14.9)	728(15.1)	450(17.2)	79887(13.5)	<0.001	373(14.7)	399(15.7)	438(17.2)	351(13.8)	0.005
Atrial fibrillation	56(1.2)	63(1.3)	31(1.2)	4487(0.8)	<0.001	29(1.1)	26(1.0)	31(1.2)	25(1.0)	0.843
CKD	41(0.9)	38(0.8)	23(0.9)	3992(0.7)	<0.001	13(0.5)	19(0.7)	23(0.9)	27(1.1)	0.154
CAD	636(13.6)	655(13.6)	343(13.1)	42962(7.2)	<0.001	318(12.5)	351(13.8)	340(13.4)	234(9.2)	<0.001
Heart failure	135(2.9)	145(3.0)	74(2.8)	10415(1.8)	<0.001	68(2.7)	77(3.0)	73(2.9)	64(2.5)	0.702
SES (%)*					<0.001					0.552
Low	1524(32.6)	1580(32.7)	782(29.9)	204822(34.5)		798(31.4)	781(30.7)	753(29.6)	766(30.2)	
High	3156(67.4)	3251(67.3)	1833(70.1)	388088(65.5)		1742(68.6)	1759(69.3)	1787(70.4)	1774(69.8)	
Urbanization (%)					<0.001					<0.001
Urban	1000(21.4)	1507(31.2)	903(34.5)	171611(28.9)		557(21.9)	827(32.6)	873(34.4)	703(27.7)	
Un-urban	3680(48.6)	3324(68.8)	1712(65.5)	421299(71.1)		1983(78.1)	1713(67.4)	1667(65.6)	1837(72.3)	
Geographic region (%)					0.004					0.064
Northern/Central	2945(62.9)	3189(66.0)	1693(64.7)	387371(65.3)		1592(62.7)	1670(65.7)	1644(64.7)	1673(65.9)	
Southern/Eastern	1735(37.1)	1642(34.0)	922(35.3)	205539(34.7)		948(37.3)	870(34.3)	896(35.3)	867(34.1)	

Abbreviation: CAD, coronary artery disease; CKD, chronic kidney disease; SES, socioeconomic status.

*SES: low: income< US$528/month, high: income ≥ US$528/month

At the end of the follow-up period, 7,774 patients had dementia (Table 2). The 6-year dementia event rates were 3.9% in the oxybutynin group, 4.3% in the solifenacin group, 2.2% in the tolterodine group and 1.2% in the control group (P<0.001). After matching, the 6-year dementia event rates after matching decreased in both oxybutynin (from 3.9% to 3.0%) and solifenacin (from 4.3% to 3.5%) users while increased by two-fold in the control group from 1.2% to 2.4%, a percentage being higher than that observed in tolderodine users (2.3%).

**Table 2:** The cumulative rate of event in difference drug in diabetes patients.

Characteristics	Before match			After match		
	n	Event (%)	p-value	n	Event (%)	p-value
Drug status			<0.001			0.035
Control	592910	7325(1.2)		2540	61(2.4)	
Oxybutynin	4680	183(3.9)		2540	77(3.0)	
Solifenacin	4831	208(4.3)		2540	88(3.5)	
Tolterodine	2615	58(2.2)		2540	58(2.3)	

**Fig 1:**
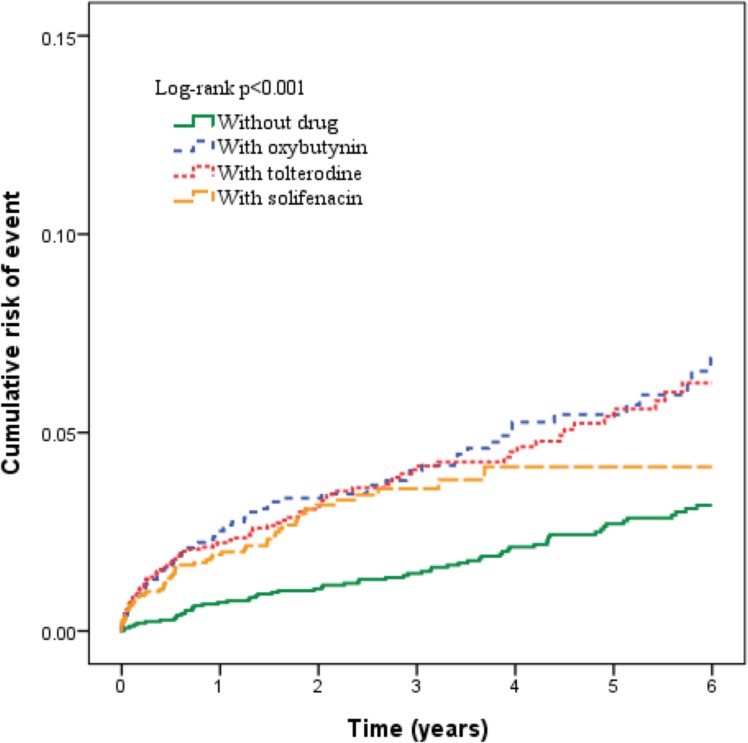
The cumulative incidence of dementia estimated by Kaplan–Meier method for patients with oxybutynin, solifenacin or tolterodine exposure

Fig 1 shows the Kaplan-Meier failure curve for the development of dementia following treatment using different anticholinergic drugs. Patients with oxybutynin, solifenacin, or tolterodine exposure were significantly more likely to develop dementia during the 6-year follow-up period (P<0.001).

The dementia HRs were significantly higher among all patients who received anticholinergic drugs. The adjusted HRs compared to nonusers of anticholinergic drugs were 2.30 (95% CI, 1.63 to 3.23), 2.26 (95% CI, 1.62 to 3.14), and 2.04 (95% CI, 1.41 to 2.95), respectively, for patients receiving oxybutynin, solifenacin, or tolterodine. S1 Table shows adjusted HRs for dementia in different anticholinergic drug exposure groups.

Table 3 shows the multivariable adjusted competing-risk regression model hazard ratios for dementia in diabetes patients receiving oxybutynin, solifenacin, or tolterodine. The competing event was death. Of the three drugs, solifenacin has the lowest relative risk for subsequent diagnosis of dementia. The adjusted HRs compared to nonusers of anticholinergic drugs were 2.35(95% CI, 1.96 to 2.81), 2.16 (95% CI, 1.81 to 2.58), and 2.24 (95% CI, 1.85 to 2.73), respectively, for patients receiving oxybutynin, solifenacin, or tolterodine. We further make a sensitivity analysis on adjusting factors (age, co-morbidity, socioeconomic status and geographic region) in S2 Table. The trend of the results remained constant.

**Table 3:** Multivariable adjusted competing-risk regression model hazard ratios of event in difference drug in diabetes patients.

Characteristics	Before match		After match	
	HR(95%CI)*	p-value	HR(95%CI)*	p-value
Drug status				
Control	1		1	
Oxybutynin	2.68(2.47–2.91)	<0.001	2.35(1.96–2.81)	<0.001
Solifenacin	2.50(2.30–2.71)	<0.001	2.16(1.81–2.58)	<0.001
Tolterodine	2.75(2.40–3.15)	<0.001	2.24(1.85–2.73)	<0.001
Age	1.07(1.07–1.07)	<0.001	1.07(1.06–1.07)	<0.001
Male	1.56(1.53–1.60)	<0.001	1.37(1.19–1.58)	<0.001
Comborbidities				
Hypertension	1.07(1.04–1.09)	<0.001	1.09(0.96–1.25)	0.200
Lipid disorders	0.78(0.76–0.81)	<0.001	0.81(0.67–0.98)	0.033
Atrial fibrillation	1.33(1.24–1.43)	<0.001	1.66(1.12–2.45)	0.012
CKD	3.29(3.10–3.49)	<0.001	2.27(1.52–3.38)	<0.001
CAD	1.05(1.02–1.09)	0.002	0.97(0.81–1.16)	0.743
Heart failure	1.40(1.34–1.48)	<0.001	1.11(0.84–1.49)	0.463
SES				
Low	1		1	
High	0.75(0.74–0.77)	<0.001	0.75(0.66–0.86)	<0.001
Urbanization				
Urban	1		1	
Un-urban	1.02(0.99–1.05)	0.093	1.05(0.91–1.23)	0.493
Geographic region				
Northern/Central	1		1	
Southern/Eastern	1.14(.11–1.17)	<0.001	1.06(0.93–1.22)	0.360

Abbreviation: HR, hazard ratio; CAD, Coronary artery disease; CI, confidence interval; CKD, Chronic kidney disease; SES, socioeconomic status.

*Adjust for the patients' age, gender, comborbidities, SES, urbanization and geographic region.

## Discussion

Our research findings show an association between taking oxybutynin, solifenacin and tolterodine and the subsequent diagnosis of dementia. Using death as a competing risk for analysis shows that, compared to DM patients with no exposure to anticholinergic drugs, those using oxybutynin, solifenacin and tolterodine respectively experience 135%, 116% and 124% increases in their hazard ratios for subsequent diagnosis of dementia.

The research subjects were all DM patients who were subsequently diagnosed with OAB and treated only using anticholinergic drugs. Typically, patients who respond well to a particular anticholinergic drug will continue to use that drug and not switch to another one. However, our research finds show that long term use of a particular anticholinergic drug may increase subsequent risk of developing dementia. This is a key finding for clinicians who must keep this potentially increased risk in mind when prescribing long-term use of a specific anticholinergic drug to DM patients suffering from OAB. Even if the patient is responding well to the specific drug, once the patient’s OAB is under control, the physician should continue therapy while simultaneously undertaking dementia prevention measures such as regular exercise, participation in social activities, and controlling body-mass index, high blood pressure, high blood sugar and high blood fat. In particular, physicians should closely monitor the cognitive function of patients on long-term anticholinergic drug regimens.

Furthermore, OAB is a debilitating condition which can significantly inhibit a patient’s ability to participate in social activities [21]. Recently developed anticholinergic drug treatments have been very successful in treating OAB, but clinicians should be mindful of the results of the present study which indicates that long-term anticholinergic drug treatment of OAB patients is associated with increased risk for dementia. Occasionally alternating treatment protocols (e.g., urination behavior therapy, intravesical drug injection, etc.) for patients who would otherwise undergo long-term oral anticholinergic drug treatment for OAB may provide safer long-term outcomes.

Another concern is that patients using anticholinergic drugs tend to be older and are more likely to suffer from other conditions including hypertension, lipid disorders, atrial fibrillation, chronic kidney disease, coronary artery disease and heart failure. Compared to DM patients not exposed to anticholinergic drugs, these patients already run a higher risk of dementia, thus clinicians must conduct risk assessment and control when putting them on long-term anticholinergic drug regimens.

This study chose DM patients as DM patients are prone to injury and are thus less suitable for participation in prospective studies. Of the many studies of the risk of dementia due to anticholinergic drug use, most focus on relatively healthy patients, and thus the results diverge from those of the present study. However, Taiwan’s national health insurance scheme covers 99.7% of Taiwan’s population and includes most illness types[16]. Thus the NHIRD provides an excellent resource for longitudinal studies, with a very low attrition rate. Thus the results of the present study can be considered highly reliable.

In addition, DM patients in Taiwan are provided with a diabetic care plan designed to enhance their quality of care[22, 23]. This “pay-for-performance” scheme provides incentives to physicians to provide consistently high-quality care and easy access. This increases the frequency of doctor visits by DM patients, which further enhances the quality of the study’s findings.

While patients suffering from cognitive impairment have a higher risk of dementia, they are not the same. Many studies of the side effects of anticholinergic drugs use cognitive impairment as their end point possibly because the onset of cognitive impairment is easier to detect over relatively short time periods[24, 25]. However, the risk of developing dementia is greater than that of cognitive impairment. Therefore the present study uses dementia as the analysis end point.

The present study suffers from certain limitations. First, we are unable to determine the severity of the patient’s OAB symptoms. However, the provision of OAB treatment is based on the degree to which symptoms impact the patient, which serves as an indicator of symptom severity.

Second, the research subjects are DM patients, without a control group of non-DM patients. Therefore, the results showing increased risk of dementia following the administration of oxybutynin, solifenacin and tolterodine cannot be extended to non-DM patients. However, the findings may provide a basis for subsequent clinical studies.

Third, trospium and darifenacin, 2 commonly used drugs for treating OAB, had not been included into this study because only 505 patients with trospium exposure more than 28cDDD and darifenacin had not been marketing in Taiwan during the enrollment period.

Forth, education and physical activity are not available in the National Health Insurance Research Database. Further studies linking administrative data are warranted. On the whole, given the magnitude and statistical significance of the effects observed in this study, this limitation is unlikely to compromise the results.

In addition, our findings show that dementia developed in only a limited number of cases, leaving us unable to further dementia types which may have to wait for future, larger-scale observations.

Finally, our determination of dementia is based on patient medical records. However, dementia is an important illness, and the reliability of medical records in recording dementia is relatively high. Moreover, the NHI conducts regular audits of patient records to improve reliability[16]. At the same time, our determination of dementia was based on diagnosis by a neurologist or psychiatrist. Moreover, to improve the quality of care provided to DM patients and reduce complications, Taiwan’s heath care system provides DM patients with personalized diabetic care plans on a pay-for-performance basis, providing incentives to physicians to ensure consistently high-quality care[22, 23]. This program improves DM patient access to healthcare and frequency of doctor visits, thus improving the likelihood that dementia will be observed and diagnosed and further improving the reliability of our findings. Therefore, while the determination of dementia is based on medical records, the reliability of this determination is very high for this DM population.

## Conclusions

In conclusion, our results find that oxybutynin, solifenacin and tolterodine increase the risk of subsequent diagnosis of dementia in DM patients, with oxybutynin having the highest increased risk of the three after using death as a competing risk for analysis. Future work will focus on the impact of these three drugs on risk of dementia in non-diabetic populations.

## Supporting information

S1 Fig Recruitment process. (TIF)

S1 Table Multivariable cox proportional hazard model hazard ratios of event in difference drug in diabetes patients. (DOCX)

S2 Table Sensitivity test. (DOCX)
