# Supplementary material for: Association between different anticholinergic drugs and subsequent dementia risk in patients with diabetes mellitus
Source: PLoS One. 2017 Apr 6;12(4):e0175335. doi: 10.1371/journal.pone.0175335 (PMC5383287; doi:10.1371/journal.pone.0175335)
Supplement: S1 Table — (DOCX) [file pone.0175335.s002.docx]

| S1 Table Multivariable cox proportional hazard model hazard ratios of event in difference drug in diabetes patients. | | | | |
| --- | --- | --- | --- | --- |
| Characteristics | Before match | | After match | |
|  | HR(95%CI)* | p-value | HR(95%CI)* | p-value |
| Drug status |  |  |  |  |
| Control | 1 |  | 1 |  |
| Oxybutynin | 3.11(2.69-3.61) | <0.001 | 2.30(1.63-3.23) | <0.001 |
| Solifenacin | 3.22(2.80-3.70) | <0.001 | 2.26(1.62-3.14) | <0.001 |
| Tolterodine | 2.98(2.30-3.86) | <0.001 | 2.04(1.41-2.95) | <0.001 |
| Age | 1.12(1.11-1.12) | <0.001 | 1.10(1.09-1.12) | <0.001 |
| Male | 0.86(0.82-0.90) | <0.001 | 0.75(0.59-0.96) | 0.022 |
| Comborbidities |  |  |  |  |
| Hypertension | 1.11(1.06-1.17) | <0.001 | 0.98(0.77-1.26) | 0.885 |
| Lipid disorders | 0.96(0.89-1.02) | 0.182 | 0.83(0.59-1.19) | 0.315 |
| Atrial fibrillation | 1.00(0.86-1.16) | 0.979 | 1.17(0.47-2.90) | 0.733 |
| CKD | 1.23(1.03-1.46) | 0.019 | 0.83(0.26-2.60) | 0.742 |
| CAD | 1.14(1.07-1.21) | <0.001 | 1.14(0.83-1.57) | 0.414 |
| Heart failure | 0.86(0.77-0.95) | 0.005 | 0.66(0.36-1.20) | 0.171 |
| SES |  |  |  |  |
| Low | 1 |  | 1 |  |
| High | 0.86(0.82-0.90) | <0.001 | 0.81(0.64-1.04) | 0.101 |
| Urbanization |  |  |  |  |
| Urban | 1 |  | 1 |  |
| Un-urban | 1.03(0.97-1.08) | 0.389 | 0.97(0.73-1.28) | 0.834 |
| Geographic region |  |  |  |  |
| Northern/Central | 1 |  | 1 |  |
| Southern/Eastern | 0.99(0.95-1.04) | 0.808 | 1.03(0.80-1.32) | 0.806 |
| Abbreviation: HR, hazard ratio; CAD, Coronary artery disease; CI, confidence interval; CKD, Chronic kidney disease; SES, socioeconomic status.  *Adjust for the patients' age, gender, comborbidities, SES, urbanization and geographic region. | | | | |
