# Supplementary material for: Association between different anticholinergic drugs and subsequent dementia risk in patients with diabetes mellitus
Source: PLoS One. 2017 Apr 6;12(4):e0175335. doi: 10.1371/journal.pone.0175335 (PMC5383287; doi:10.1371/journal.pone.0175335)
Supplement: S2 Table — (DOCX) [file pone.0175335.s003.docx]

S2 Table. Sensitivity test: Multivariable adjusted competing-risk regression model hazard ratios of event in difference drug in diabetes patients.

| Characteristics | Scenario 1 | Scenario 2 | Scenario 3 | Scenario 4 |
| --- | --- | --- | --- | --- |
|  | HR(95%CI) | HR(95%CI) | HR(95%CI) | HR(95%CI) |
| Drug status |  |  |  |  |
| Control | 1 |  | 1 | 1 |
| Oxybutynin | 2.30(1.92-2.76)*** | 2.32(1.94-2.78) *** | 2.37(1.98-2.84) *** | 2.37(1.98-2.83) *** |
| Solifenacin | 2.09(1.76-2.50)*** | 2.15(1.81-2.57) *** | 2.16(1.81-2.57) *** | 2.16(1.81-2.58) *** |
| Tolterodine | 2.20(1.81-2.68)*** | 2.25(1.85-2.74) *** | 2.23(1.84-2.72) *** | 2.24(1.84-2.72) *** |
| Age |  | 1.07(1.06-1.08) *** | 1.07(1.06-1.08) *** | 1.07(1.06-1.07) *** |
| Male | 1.59(1.38-1.82)*** | 1.40(1.21-1.60) *** | 1.37(1.19-1.57) *** | 1.36(1.18-1.57) *** |
| Comborbidities |  |  |  |  |
| Hypertension | 1.40(1.23-1.60)*** |  | 1.09(0.96-1.25) | 1.09(0.96-1.25) |
| Lipid disorders | 0.74(0.61-0.90) ** |  | 0.81(0.67-0.98) * | 0.81(0.67-0.98) * |
| Atrial fibrillation | 1.77(1.19-2.63) ** |  | 1.66(1.12-2.45) * | 1.66(1.12-2.46) * |
| CKD | 2.89(1.95-4.29)*** |  | 2.35(1.58-3.50) *** | 2.29(1.54-3.41) *** |
| CAD | 1.19(0.99-1.43) |  | 0.99(0.83-1.19) | 0.97(0.81-1.16) |
| Heart failure | 1.61(1.21-2.15)*** |  | 1.11(0.83-1.48) | 1.12(0.84-1.49) |
| SES |  |  |  |  |
| Low | 1 | 1 |  | 1 |
| High | 0.63(0.55-0.71)*** | 0.75(0.66-0.85) *** |  | 0.76(0.67-0.87) *** |
| Urbanization |  |  |  |  |
| Urban | 1 | 1 | 1 |  |
| Un-urban | 1.18(1.01-1.37) * | 1.08(0.93-1.26) | 1.01(0.87-1.18) |  |
| Geographic region |  |  |  |  |
| Northern/Central | 1 | 1 | 1 |  |
| Southern/Eastern | 1.09(0.95-1.24) | 1.07(0.94-1.22) | 1.05(0.92-1.20) |  |

Abbreviation: HR, hazard ratio; CAD, Coronary artery disease; CI, confidence interval; CKD, Chronic kidney disease; SES, socioeconomic status.

Scenario 1: Adjust for the patients' gender, comborbidities, SES, urbanization and geographic region.

Scenario 2: Adjust for the patients' age, gender, SES, urbanization and geographic region.

Scenario 3: Adjust for the patients' age, gender, comborbidities, urbanization and geographic region.

Scenario 4: Adjust for the patients' age, gender, comborbidities, SES..

* p<0.05, ** P<0.01, *** p<0.001
